# Effectiveness and acceptability of methods of communicating the results of clinical research to lay and professional audiences: protocol for a systematic review

**DOI:** 10.1186/s13643-019-1065-x

**Published:** 2019-06-25

**Authors:** Annabelle South, Julia Bailey, Mahesh K. B. Parmar, Claire L. Vale

**Affiliations:** 1 MRC Clinical Trials Unit at UCL and Department of Primary Care and Population Health, UCL, ICTM, 2nd Floor, 90 High Holborn, London, WC1V 6LJ UK; 2 Department of Primary Care and Public Health, UCL, London, UK; 3 MRC Clinical Trials Unit at UCL, London, UK

**Keywords:** Communication, Dissemination, Clinical studies, Patients, Medical professionals, Policymakers

## Abstract

**Background:**

Phase III randomised controlled trials aim not just to increase the sum of human knowledge, but also to improve treatment, care or prevention for future patients through changing policy and practice. To achieve this, the results need to be communicated effectively to several audiences. It is unclear how best to do this while not wasting scarce resources or causing avoidable distress or confusion. The aim of this systematic review is to examine the effectiveness, acceptability and resource implications of different methods of communication of clinical research results to lay or professional audiences, to inform practice.

**Methods:**

We will systematically review the published literature from 2000 to 2018 for reports of approaches for communicating clinical study results to lay audiences (patients, participants, carers and the wider public) or professional audiences (clinicians, policymakers, guideline developers, other medical professionals). We will search Embase, MEDLINE, PsycINFO, ASSIA, the Cochrane Database of Systematic Reviews and grey literature sources. One reviewer will screen titles and abstracts for potential eligibility, discarding only those that are clearly irrelevant. Potentially relevant full texts will then be assessed for inclusion by two reviewers. Data extraction will be carried out by one reviewer using EPPI-Reviewer. Risk of bias will be assessed using the relevant Cochrane Risk of Bias 2.0 tool, ROBINS-1, AXIS Appraisal Tool or Critical Appraisals Skills Programme Qualitative Checklist, depending on study design. We will decide whether to meta-analyse data based on whether the included trials are similar enough in terms of participants, settings, intervention, comparison and outcome measures to allow meaningful conclusions from a statistically pooled result. We will present the data in tables and narratively summarise the results. We will use thematic synthesis for qualitative studies.

**Discussion:**

Developing the search strategy for this review has been challenging as many of the concepts (patients, clinicians, clinical studies, and communication) are widely used in literature that is not relevant for inclusion in our review. We expect there will be limited comparative evidence, spread over a wide range of approaches, comparators and populations and, therefore, do not anticipate being able to carry out meta-analysis.

**Systematic review registration:**

International Prospective Register of Systematic Reviews PROSPERO (CRD42019137364).

**Electronic supplementary material:**

The online version of this article (10.1186/s13643-019-1065-x) contains supplementary material, which is available to authorized users.

## Background

Phase III randomised controlled trials are often costly and can take years to carry out [1]. They may involve hundreds or thousands of participants, cared for by clinicians, nurses and other medical professionals at many sites. They aim not just to increase the sum of human knowledge, but also to improve treatment, care or prevention for future patients. To achieve this, the results need to be communicated effectively to a variety of audiences [2].

The evidence base on how best to communicate trial results to different audiences is sparse [2]. These gaps in evidence mean that scarce time and resources may be wasted in carrying out ineffective communications activities, while approaches that do work may not be widely used. There is also a risk that some communication approaches may be harmful, for example causing avoidable distress or confusion [3, 4]. This systematic review will draw together what evidence there is, in order to inform the practice of clinical trials units and others who are interested in effectively communicating trial results.

The aim of this study is to examine the effectiveness, acceptability and resource implications of different methods of communication of clinical research results to lay (study participants, patients, carers, communities, populations at risk of the condition, and the wider public) and professional audiences (medical professionals, policymakers, clinical guideline developers and healthcare commissioners). We are primarily interested in evidence on communicating the overall results of phase III academic clinical trials but will also look at the literature on communicating the results of other clinical research study designs that are likely to generate evidence with direct implications for policy and practice (for example, systematic reviews and cohort studies) in order to learn lessons that may also be applicable to phase III academic clinical trials. We will try to encompass all approaches to communication that have been evaluated in the literature.

This systematic review will build on two prior reviews of communicating the results of clinical research, identified through scoping searches of the PubMed database during the initial planning phase of this review. The first of these was a systematic review by the Agency for Healthcare Research and Quality (AHRQ) [2], which looked at several questions around the communication and dissemination strategies to facilitate the use of health-related evidence. Of most relevance to this review is the comparative effectiveness of the dissemination strategies to promote the use of health care evidence and how this varies by patients and clinicians. They focused on strategies to increase reach of information, motivation to use and apply evidence, ability to use and apply evidence, or used a multicomponent approach (combining two or more approaches to increase the reach of the evidence, the motivation of the audience to apply the information, and/or the ability to use the evidence). They found that evidence was poor, inconsistent or not statistically significant for most of the comparisons they looked at. The most successful strategy identified in the review was the use of a multicomponent dissemination approach for clinicians when trying to change their behaviours. This review will complement the AHRQ review by including qualitative and nonexperimental studies alongside trials of communication approaches, to provide a broader understanding of approaches that are being used to communicate health research, as well as look at comparative evidence that has been generated since the AHRQ review. The second literature review looked at communicating the results of clinical research to participants [5] and found that research participants wanted to be informed of the results of the studies they had taken part in and that investigators seemed to support the communication of aggregate results to participants. Our scoping search of the PROSPERO database did not reveal any other relevant systematic reviews to consider.

Our review will also complement a study currently being carried out to look at communicating results to trial participants (the REporting Clinical trial results Appropriately to Participants (RECAP) study) [6]. In addition to study participants, we will also look at communicating results to wider lay audiences (including patients who are not participating in the trial, and the wider public) and professional audiences including policymakers, guideline developers and clinicians.

This review will cover a broad range of approaches of communicating study results to lay and professional audiences. As there are very different resource implications for the different methods of communicating results, this review will compare the effectiveness and acceptability of different methods and will also comment on resource implications. It will look at a variety of outcomes, covering all dimensions of the International Association for the Measurement and Evaluation of Communication Framework (outputs, outtakes (what the audience take from the communication, e.g. awareness or understanding), outcomes and impact) [7]. Where there is sufficient evidence, we will seek to make recommendations on which approaches are likely to be the most effective, cost-effective and/or acceptable for communicating with different audiences, in order to guide future practice and avoid effort and resources being wasted on ineffective approaches.

## Objectives

Our overarching research question is what are the best ways or combinations of ways of communicating the results of clinical research that has implications for health policy or practice to lay and professional audiences? Within this, we will look at three key questions:

1. How effective are different approaches to communicating the results of clinical research to:
  - Clinicians (beyond those that were involved in the study)
  - Policymakers, guideline developers and healthcare commissioners
  - Study participants and their carers
  - Other lay audiences, including patients and the public?
2. What factors influence the effectiveness of different approaches to communicating the results of clinical research?
3. What are the views and experiences of different audiences about how the results of clinical studies are communicated to them, and what are the views and experiences of those involved in communicating the results of clinical studies to these audiences around how this is done?

## Methods

This protocol will be reported according to the PRISMA-P statement. Our PRISMA-P checklist is included as Additional file 1.

### Eligibility criteria

Studies will be selected according to the criteria outlined below.

#### Study designs

We will include any reports of studies with any qualitative or quantitative study design, as well as theoretical papers, health economic papers, reviews, reports and guidelines.

#### Population

Eligible participants will include lay audiences and professional audiences, as defined below.

##### Lay audiences

- Clinical study participants and their carers, the wider patient community, including individual patients, carers, and patient groups
- Communities in which the studies have taken place (geographic or other demographic communities)
- Populations at risk of the condition
- The wider public

##### Professional audiences

- Medical professionals (beyond those involved in conducting the trial), including individual practitioners, organisations (e.g. hospitals or medical schools) and professional associations/societies
- Policymakers
- Clinical guideline developers
- Healthcare commissioners

We will not restrict the population by age, sex, location or other demographic factors.

#### Approaches

For the purposes of this review, we are interested in any approaches for communicating study results to any of the populations specified above. We are interested in communicating the results of clinical studies carried out on humans that have implications for health policy or practice. By clinical studies, we mean observational or interventional medical research relating to treatment, diagnosis or disease prevention among actual patients/people, rather than laboratory or modelling studies. We anticipate that this will include approaches to communicating the results of phase III academic randomised controlled trials, including cluster randomised trials; meta-analyses; epidemiological studies that look at treatment, prevention or diagnosis approaches; industry-sponsored phase III randomised controlled trials; and possibly phase II randomised controlled trials if their results have implications for practice and/or policy. As we will include studies without a comparator group in this systematic review, we are not restricting the search to studies with a particular comparator.

#### Outcomes

For question 1 (effectiveness), we are interested in a broad range of outcomes (Table 1), so the types of outcomes reported will not be an inclusion or exclusion criterion.

**Table 1:** Outcomes we expect to find information on, by audience

	Outputs	Outtakes	Outcomes	Impact
Lay audiences	Demand uptake/reach	Awareness Knowledge *Understanding*/comprehension/clarity Reaction to the results	Attitude to the results (interest, consideration) *Satisfaction with how the results have been communicated*/*the information provided* Satisfaction with taking part in the study	Willingness to take part in future research Reported likelihood of recommending that others take part in research Changes in behaviour based on results
Professional audiences	Demand uptake/reach	Awareness Knowledge Understanding/comprehension/clarity	Attitude to the results (e.g. interest, consideration, support)	*Changes in practice* *Changes in policy* *Changes in guidelines*
Research communicators	Practice *costs*	Barriers to communicating results Facilitators to communicating results		

#### Timing

The time point of enrolment and the duration of interventions will not be considered as eligibility criteria. There will be no restrictions by length of follow-up of outcomes.

#### Setting

We will not restrict the search to specific settings but will collect data on the settings in which studies have been carried out and seek to assess whether there is heterogeneity of effect based on setting.

#### Minimum sample size

We will not restrict studies by sample size, as this would be inappropriate when including qualitative research, or studies where the intended audience may be small (e.g. policymakers).

#### Language

We will include articles reported in English. We will exclude articles written in other languages, due to resource constraints.

#### Types of publication

The types of publications we will include are:

- Complete articles
- Conference abstracts
- Reports (grey literature)
- Theses

We will exclude commentaries, editorials, guidelines, letters and protocols.

### Information sources

Literature search strategies will be developed using medical subject headings (MeSH) and text words. We will search the following databases:

- Embase (Ovid interface)
- MEDLINE (Ovid interface)
- PsycINFO (Ovid interface)
- ASSIA
- Cochrane Database of Systematic Reviews

We will also search the following grey literature sources for items:

- ProQuest Dissertations and Theses Global
- The INVOLVE evidence library
- The National Coordinating Centre for Public Engagement’s resources
- Conference proceedings from the Society of Clinical Trials’ annual meetings
- Conference proceedings from the International Clinical Trials Methodology Conference
- Conference proceedings from the Engage conference
- Cochrane Colloquium abstracts

We will search these databases and information sources for records from January 1, 2000, onwards. We have chosen this time period as there have been considerable developments in the use of communication technologies since 2000 (e.g. email, internet and mobile phones), which may have implications for the communication of study results. In addition, a scoping search revealed that very little had been published on this topic prior to 2000, which supports this decision.

We will scan the reference lists of key included studies and relevant reviews and guidelines identified through the search, to identify papers that our search has missed. We will contact experts in the field, to ask what studies they are aware of that we should make sure we include.

### Search strategy

No study design limits will be imposed on the search. The search strategy was developed by AS, who is a PhD student, under the guidance of CV, who is a specialist in systematic reviews, with advice from an information specialist. AS, CV and JB contributed to the development of search terms and reviewed the final strategy. The strategy was developed and pilot tested in the EMBASE database, using the Ovid interface, using an iterative approach until a strategy that was sufficiently sensitive but did not result in an unmanageable number of records was achieved. The strategy will be adapted to the syntax and subject headings of other databases, including MEDLINE, PsycINFO and ASSIA as necessary.

Some of the grey literature sources have less sophisticated search functions. For these, we will use broad search terms, and hand search the results to identify those that are potentially relevant for this review. The searches will be updated until the study completion date, which is anticipated to be around early 2022.

**Fig. 1:**
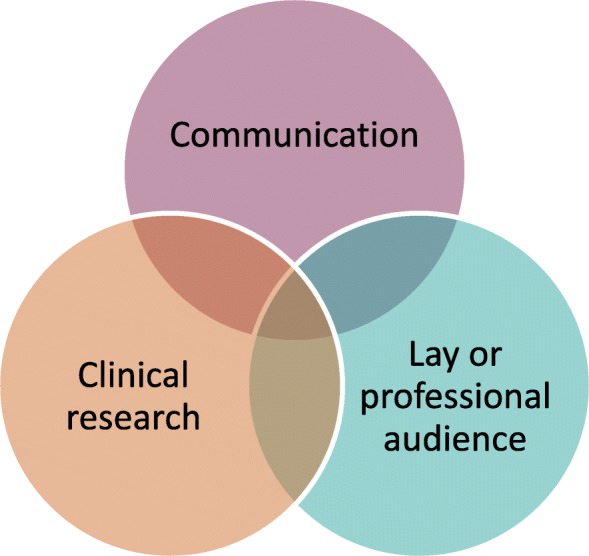
Concepts of the search strategy

Two searches will be run: one to identify articles relevant to communicating results to lay audiences and the other to identify articles relevant to communicating results to professional audiences. This is because a different terminology is needed to identify communication approaches for professional audiences compared to lay audiences. Figure 1 illustrates the concepts of our search. Each search will combine results from searches focusing on terms for the audience, with results from searches with terms for communication and clinical research. Additional file 2 shows the list of search terms used. These will be combined with adjacency rules applied to limit results to articles where the audience terms, communication terms and clinical research terms are within a certain number of words from each other. Where databases allow us to use this adjacency approach, we will not use MeSH terms in our searches, as we are confident that articles that are relevant to our review will be identified through use of terms relating to audience, communications and clinical research in their titles, abstracts and keywords, and it is unclear how keywords about patients or clinical research are applied, given how common these are in the medical literature. Where the adjacency approach cannot be used, we will use MeSH terms for communication to narrow down results.

### Study records

#### Data management

Search results will be downloaded into Endnote. The citations will then be imported into Covidence (www.covidence.org) for abstract and title screening. Covidence will be used to record eligibility decisions. Selected records will then be imported into EPPI-Reviewer Version 4.7.0.0 [8] for data extraction.

#### Selection process

AS will assess the eligibility of titles and abstracts identified from electronic searches against the eligibility criteria of the review, discarding only those that are duplicates or clearly irrelevant. She will retrieve full-text copies of all articles judged to be potentially eligible. AS and CV will then independently assess all retrieved articles for inclusion. AS and CV will meet regularly during the screening process to discuss any issues arising and to ensure the eligibility criteria are being applied consistently. In cases where the two reviewers disagree on whether an item is eligible, this will be resolved by discussion between AS, CV and a third author where necessary. Where there is insufficient information in the publication to assess its eligibility, we will seek additional information from the study authors. All potentially relevant papers excluded from the review at this stage will be listed as excluded studies, with reasons provided in the characteristics of excluded studies table. The reviewers will not be blind to the journal titles or to the study authors or institutions.

We will not use quality criteria as an inclusion criterion, but will assess the quality of included studies and report on that in our synthesis. If we find many records, we may restrict the synthesis to those judged to be at low risk of bias. See the “Assessment of risk of bias in included studies” section for how we will assess the quality of studies.

#### Data collection process

AS will carry out data extraction using standard electronic forms to tabulate the required information in EPPI-Reviewer. This will include information about the study design, setting, results to be communicated, communication approach(es) used, target audience and outcomes. The form will be based on the Cochrane Consumers and Communication Group’s data extraction template, adapted to fit the needs of this review. There will be different versions of the form for quantitative and qualitative studies, to allow different risk of bias assessment tools to be applied and different types of data to be recorded. Draft data collection forms are included as Additional file 3. The forms will be piloted on the first 5 studies of each type and adapted as needed. If the piloting leads to changes to our plans, we will be transparent about this when we report the review. Where there are multiple reports from a single study, the most recently published data on each available outcome will be recorded. If important data about outcomes or approaches are missing, we will contact authors to request this information.

### Data items

We will collect information about:

- The article type, publication year, citation and contact details for authors
- The study it refers to communicating the results of (design, population, setting, disease, time period and funding)
- The study methods for this report
- The participants included
- The approaches to communications studied
- The outcome measures
- The results/findings and interpretation
- Risk of bias

### Outcomes and prioritisation

We will classify outcomes in line with the International Association for Measurement and Evaluation of Communication (AMEC) framework [7], which splits measurements and insights into outputs, outtakes (the response and reactions of the audience to the activity), outcomes (the effect of the communication on the target audience) and impact (changes the audience make as a result of the communication). Table 1 outlines the outcomes that we expect to find information on (although they will not all be relevant for every study). We may find additional outcomes that fit within this framework. Our primary outcomes are shown in italics. For participants, the co-primary outcomes are understanding and satisfaction with how the results are communicated. For other patients, communities and the public, we have chosen understanding as our primary outcome. For clinicians and policymakers, our co-primary outcomes are changes in the recommendations made in clinical policy documents, and clinical guidelines published by professional bodies or government agencies, and changes in clinical practice. We are also interested in the costs of the approaches tested. We will not set time points for measuring primary or secondary outcomes but use those reported in eligible studies.

The qualitative synthesis will focus on research question 2 (factors that influence the effectiveness of different communication approaches) and 3 (the views and experiences of audiences and communicators with regard to the communication of research results).

The AHRQ review reported a lack of consensus regarding definitions of key terms, particularly those describing different dissemination strategies. As there are no widely agreed standardised approaches to measuring the outcomes listed in Table 1, we will accept different definitions and approaches to the measurement of outcomes, as reported in retrieved studies, but will note how they have been defined by the authors.

### Assessment of risk of bias in included studies

We will apply the risk of bias tool according to the pertinent study design. If any RCTs of communication approaches are found, we will use the Cochrane ‘RoB 2.0’ tool [9]. Any cluster randomised trials will be assessed by the Cochrane ‘R0B 2.0 for cluster randomised trial when interest is in the effect of assignment to intervention’ template [9]. Cohort studies and case-control studies will be assessed using the ROBINS-I tool [10]. Cross-sectional studies will be assessed using the AXIS tool [11]. Qualitative papers selected for inclusion will be assessed for methodological quality using the CASP Qualitative Checklist [12], which is one of the tools recommended by the Cochrane Qualitative and Implementation Methods Group for assessing the quality of qualitative studies with a range of methods [13]. For other types of studies, the most appropriate tool for assessing risk of bias will be applied.

### Data synthesis

#### Narrative synthesis

We will group the data based on the category that best explores the heterogeneity of studies and makes the most sense to the reader (i.e. by interventions, populations or outcomes). Within each category, we will present the data in tables and narratively summarise the results. Information will be presented in the text and tables to summarise the characteristics and findings of the included studies. The narrative synthesis will explore the relationship and findings both within and between the included studies. We will use thematic synthesis for qualitative studies.

Results will be presented in order of key question, and, within the section on key question 1, we will present the primary outcomes first, followed by the other outcomes in the order of outputs, outtakes, outcomes and impact.

If we find many studies relating to a communication approach to similar audiences, we may exclude from the synthesis those at high risk of bias. If we do not find large numbers of comparable studies, we will retain studies of any level of risk of bias in our analyses, but ensure that the narrative considers the impact that the level of risk of bias has on the certainty of any conclusions drawn. If there is sufficient information available, we will seek to explore whether the effectiveness of communication approaches varies by the target audience (e.g. lay vs professional audiences, participants vs other patients, patients vs general population), disease or geographical location, or other factors identified from the qualitative synthesis.

For qualitative studies, we will accompany the narrative synthesis with a summary of the assessment of quality for the included studies and how methodological limitations may affect our confidence in the synthesised findings, as recommended by the Cochrane Qualitative and Implementation Methods Group [13].

Following the GRADE guidelines, we will grade the overall quality of the synthesised quantitative evidence for each outcome separately as high, moderate, low or very low, taking into account the risk of bias, effect size, consistency of results, directness of evidence, precision and risk of publication bias [14].

#### Meta-analysis

We do not think that formal meta-analyses will be possible because of the anticipated variability in the populations, interventions and outcomes of the included studies. However, we will conduct such quantitative syntheses in the case of (1) low risk of bias in the included studies, (2) consistent outcomes between studies, (3) low publication bias, (4) a high number of included studies and (5) low heterogeneity [15, 16]. We will apply the random effects model when undertaking a meta-analysis. If we are unable to pool the data statistically using meta-analysis, we will provide clear reasons for this decision.

### Meta-biases

We will carry out extensive literature searches that include the grey literature as well as published studies in order to limit the impact of publication bias on our review. We will also make careful assessments of potential multiple publications from a single study, to ensure we are not double counting results. We will also ask key stakeholders what they think are the key studies in this area, to ensure our search has identified them.

Where protocols for studies have been published, we will check for differences between the protocol and the final study, to assess whether there has been selective outcome reporting. Where insufficient information is available from the published report, we will contact the authors for further information.

## Discussion

Through conducting this review, we hope to bring together the existing evidence on how best to communicate the results of clinical research to lay and professional audiences, in terms of effectiveness, acceptability and resource requirements, in order to inform practice.

Developing the search strategy for this study has been challenging as many publications that are unrelated to our topic use combinations of the search terms and concepts used (e.g. participants, clinicians, clinical studies, report, disseminate), resulting in very large numbers of records if our three concepts (lay or professional, communication and clinical research) are combined with AND. The adjacency approach has been developed to improve the specificity of results from the searches, with the number of words within which the three concepts have to appear being determined based on comparing results from different limits (e.g. adjacent within 5 words compared to adjacent within 6 words), to see at what point adding extra words identifies very few extra relevant records.

Previous reviews, looking at subsets of the audiences we are interested in, have found very heterogeneous studies in terms of interventions and outcome measures, making meta-analysis challenging. As our review is looking at a wide range of audiences, interventions and outcomes, we expect that we may not be able to carry out meta-analyses. Once we have carried out the screening and data extraction, we will make a decision on whether meta-analysis is appropriate.

A limitation of this review is that title and abstract screening will be carried out by only one reviewer, which could result in potentially relevant records being excluded. In order to reduce this risk, the single reviewer will include any records where there is doubt about their eligibility at this stage of the review.

## Additional files

Additional file 1: PRISMA-P+ checklist. Completed PRISMA-P checklist for this protocol (DOCX 36 kb)

Additional file 2: Search terms. List of search terms used in the search strategy. (DOCX 17 kb)

Additional file 3: Draft data collection forms. Draft data collection forms. (DOCX 72 kb)

## Data Availability

Data sharing is not applicable to this article as no datasets were generated or analysed to develop this protocol. Once the review has been completed, data will be available following the MRC Clinical Trials Unit at UCL’s data sharing policy https://www.ctu.mrc.ac.uk/our-research/other-research-policy/data-sharing/.

## Abbreviations

AHRQ: Agency for Healthcare Research and Quality
AMEC: International Association for Measurement and Evaluation of Communication
ASSIA: Applied Social Science Index & Abstracts
CASP: Critical Appraisal Skills Programme
ICTM: Institute of Clinical Trials and Methodology
MeSH: Medical subject headings
MRC: Medical Research Council
RECAP: REporting Clinical trial results Appropriately to Participants
RoB: Risk of bias
UCL: University College London
