# Supplementary material for: Effectiveness and acceptability of methods of communicating the results of clinical research to lay and professional audiences: protocol for a systematic review
Source: Syst Rev. 2019 Jun 25;8:150. doi: 10.1186/s13643-019-1065-x (PMC6593506; doi:10.1186/s13643-019-1065-x)
Supplement: Supplementary file 2 — Search terms. List of search terms used in the search strategy. (DOCX 17 kb) [file 13643_2019_1065_MOESM2_ESM.docx]

# Additional File 2: Search terms

Keyword terms for audiences

- Participant*
- Volunteer*
- patient*
- subject*
- consumer*
- caregiver*
- carer*
- relative*
- relation*
- public
- communit*
- lay audience*
- "policy maker*"
- policymak*
- "decision maker"
- commissioner*
- "guideline develop*"
- "health care professional*"
- "health care provider*"
- "health care worker*"
- "health personnel"
- "health professional*"
- "health profession personnel"
- "healthcare personnel"
- "healthcare professional*"
- "healthcare practitioner*"
- "healthcare provider*"
- "healthcare worker*"
- healthworker*
- clinician*
- doctor*
- "medical professional*"
- "medical personnel"
- nurse*
- "health professional*"
- "clinical community"
- professional*
- physician*
- "medical practitioner*"
- practitioner*

Keyword terms for communication

- "medical information"
- "health communication"
- communicat*
- inform*
- offer*
- disclos*
- return*
- "feeding back"
- feedback
- "feed back"
- provid*
- (deliver* adj4 result*)
- shar*
- notif*
- (disseminat* adj4 result*)
- (presenting adj4 result*)
- (reporting adj4 result*)
- "information dissemination"
- "clinical decision making"
- "family decision making"
- "medical decision making"
- "patient decision making"
- "shared decision making"
- "lay summary"
- "patient education"
- "plain English"
- "persuasive communication"
- "health education"
- "social marketing"
- "public engagement"
- "mass communication"
- "health communication"
- "medical information"
- "information dissemination"
- "dissemination strateg*"
- "information service*"
- "academic detailing"
- guideline*

MeSH terms for communication

- medical information/
- information dissemination/
- clinical decision making/ or family decision making/ or medical decision making/ or patient decision making/ or shared decision making/
- patient education/
- persuasive communication/
- health education/
- social marketing/
- mass communication/
- information service/

Keyword terms for clinical research

- "clinical research"
- “clinical study”
- "cohort study"
- "clinical trial*"
- "meta-analys*"
- "systematic review"
- "epidemiological stud*"
- "randomi* controlled trial"
- "observational study"
- trial
- (clinical adj2 study)
