# Supplementary material for: Effectiveness and acceptability of methods of communicating the results of clinical research to lay and professional audiences: protocol for a systematic review
Source: Syst Rev. 2019 Jun 25;8:150. doi: 10.1186/s13643-019-1065-x (PMC6593506; doi:10.1186/s13643-019-1065-x)
Supplement: Supplementary file 3 — Draft data collection forms. Draft data collection forms. (DOCX 72 kb) [file 13643_2019_1065_MOESM3_ESM.docx]

# Additional File 3: Data Collection Forms

## Form 1: Data collection form for quantitative studies

### Section 1: General information

| Date form completed: |  |
| --- | --- |
| Study ID: |  |
| Citation: |  |
| Year: |  |
| Article type: |  |
| Author contact details for study: |  |
| Further information required: |  |
| Correspondence with authors: |  |
| Any additional unpublished data supplied? |  |
| Other information & notes |  |

### Section 2: Study it refers to

|  | **Instructions** | **Data** |
| --- | --- | --- |
| Type of study | *Eg. RCT, cohort, systematic review. If RCT, describe phase and design.* |  |
| Study disease area |  |  |
| Geographical location |  |  |
| Study population | *Describe age, gender, ethnicity and other key characteristics that may be relevant* |  |
| Study interventions | *If applicable* |  |
| Number of participants | *By group, if applicable* |  |
| When study took place |  |  |
| Funding source |  |  |
| Headline finding |  |  |

### Section 3: Study methods

| Aim of study |  |
| --- | --- |
| Study design |  |
| Number of groups & description |  |
| Data collection methods |  |
| Data analysis methods |  |

### Section 4: Participants in this study of communications

|  | **Instructions** | **Data** |
| --- | --- | --- |
| Who data was collected from | *Description. Professional or lay?* |  |
| Geographic location | *Where this study of communications took place* |  |
| Setting | *Where this study of communications took place.* |  |
| Method of recruitment of participants |  |  |
| Age: range, mean |  |  |
| Gender |  |  |
| Ethnicity |  |  |
| Number of participants approached |  |  |
| Number who refused to take part |  |  |
| Number excluded |  |  |
| Randomised to each group |  |  |
| Withdrawn | *By group, if appropriate* |  |
| Lost-to-follow-up | *By group, if appropriate* |  |
| Included in analysis | *By group, if appropriate* |  |

### Section 5: Approach to communications studied

|  | **Instructions** | **Approach** | **Control (if applicable)** |
| --- | --- | --- | --- |
| Approach name |  |  |  |
| Approach aim & rationale | *Describe any theory and evidence base supporting the approach* |  |  |
| Tool(s)/Materials | *Describe the content, format(s) or media, source of materials (if possible, where they can be accessed), and any other information relevant to the physical or information materials provided to participants or in training providers of the approach.* |  |  |
| Procedures |  |  |  |
| Co-interventions |  |  |  |
| Mode of delivery | *eg. face-to-face / distant; individual/group* |  |  |
| Who delivered the communication? | *Include description of any specific training given to providers to deliver the communication, numbers of providers, professional background, specific pre-existing skills or experience required, quality of any specific training received to deliver the communication, and any measures of competence or consistency in delivering the communication recorded before or during the study.* |  |  |
| Where was communication provided | *(eg country, type of clinic, primary or hospital care).* |  |  |
| When and how often or much of the communication approach was provided? | *Describe how the approach was delivered, such as stages, timing, frequency, number of sessions, intensity and duration of communication delivery.* |  |  |
| Was the communication approach tailored? | *If the communication approach was meant to be tailored or personalised in the course of the study, describe the rationale for this and the major features of what was done - such as:*   - *how?* - *why?* - *when? and* - *what?*   *was done to tailor the approach.*  *If particular decision rules were used to determine when or how to tailor the approach details should be provided.* |  |  |
| Was the approach modified or adapted? | *If the approach was changed during the study, this should be described* |  |  |
| How well was the approach delivered? | *Assessment of fidelity: if approach fidelity was assessed, describe the extent to which the approach was delivered as intended.* |  |  |

### Section 6: Outcome measures

| **Primary outcomes** | | | |
| --- | --- | --- | --- |
| **Outcome** (including definition provided by authors) | **Method of assessing outcome measures**  *eg, phone survey, questionnaire* | **Method of follow-up for non-respondents** | **Timing of outcome assessment**  *(including frequency, length of follow up)* |
|  |  |  |  |
|  |  |  |  |

| **Primary outcomes - adverse events**  (*eg complaints, levels of dissatisfaction, adverse incidents, side effects, increased inequities)* | | |
| --- | --- | --- |
| **Adverse event** (including definition provided by authors) | **Method of assessment** | **Timing of assessment** |
|  |  |  |
|  |  |  |
|  |  |  |

| **Secondary outcomes** | | | |
| --- | --- | --- | --- |
| **Outcome**  (including definition provided by authors) | **Method of assessing outcome measures**  *eg, phone survey, questionnaire* | **Method of follow-up for non-respondents** | **Timing of outcome assessment**  *(including frequency, length of follow up)* |
|  |  |  |  |
|  |  |  |  |
|  |  |  |  |
|  |  |  |  |
|  |  |  |  |

### Section 7: Data and Results

#### Dichotomous outcomes

| **Outcome** | **Timing of outcome assessment (days/months)** | **Approach group*** | | **Control group** | | **Notes** |
| --- | --- | --- | --- | --- | --- | --- |
|  |  | **Observed (n)** | **Total (N)** | **Observed (n)** | **Total (N)** |  |
|  |  |  |  |  |  |  |
|  |  |  |  |  |  |  |
|  |  |  |  |  |  |  |
|  |  |  |  |  |  |  |
|  |  |  |  |  |  |  |
|  |  |  |  |  |  |  |

**Note: add additional columns if there is more than one intervention group, eg. Intervention Group A, Intervention Group B…*

#### Continuous outcomes

| **Outcome** | **Timing of outcome assessment (days/months)** | **Intervention group** | | | **Control group** | | | **Notes** |
| --- | --- | --- | --- | --- | --- | --- | --- | --- |
|  |  | ***Mean / Mean change** | **Standard deviation** | **N** | ***Mean / Mean change** | **Standard deviation** | **N** |  |
|  |  |  |  |  |  |  |  |  |
|  |  |  |  |  |  |  |  |  |
|  |  |  |  |  |  |  |  |  |
|  |  |  |  |  |  |  |  |  |
|  |  |  |  |  |  |  |  |  |
|  |  |  |  |  |  |  |  |  |

**delete as appropriate*

#### Other results or data

| **Outcome** | **Timing of outcome assessment** | **Intervention Group** | **Control group** | **Notes** |
| --- | --- | --- | --- | --- |
|  |  |  |  |  |
|  |  |  |  |  |
|  |  |  |  |  |

### Section 8: Risk of bias

*Complete the relevant Risk of Bias form for the study type*

#### Risk of bias assessment for a parallel group trial with interest in the effect of assignment to intervention^[[1]](#footnote-1)^

| **Domain** | **Signalling questions** | **Response options** | **Description/Support for judgement** |
| --- | --- | --- | --- |
| **Bias arising from the randomization process** | 1.1 Was the allocation sequence random? | Y / PY / PN / N / NI |  |
|  | 1.2 Was the allocation sequence concealed until participants were recruited and assigned to interventions? | Y / PY / PN / N / NI |  |
|  | 1.3 Were there baseline imbalances that suggest a problem with the randomization process? | Y / PY / PN / N / NI |  |
|  | **Risk of bias judgement** | Low / High / Some concerns |  |
|  | Optional: What is the predicted direction of bias arising from the randomization process? | Favours experimental / Favours comparator / Towards null /Away from null / Unpredictable |  |
| **Bias due to deviations from intended interventions** | 2.1. Were participants aware of their assigned intervention during the trial? | Y / PY / PN / N / NI |  |
|  | 2.2. Were carers and trial personnel aware of participants' assigned intervention during the trial? | Y / PY / PN / N / NI |  |
|  | 2.3. If Y/PY/NI to 2.1 or 2.2: Were there deviations from the intended intervention beyond what would be expected in usual practice? | NA / Y / PY / PN / N / NI |  |
|  | 2.4. If Y/PY to 2.3: Were these deviations from intended intervention unbalanced between groups *and* likely to have affected the outcome? | NA / Y / PY / PN / N / NI |  |
|  | 2.5 Were any participants analysed in a group different from the one to which they were assigned? | Y / PY / PN / N / NI |  |
|  | 2.6 If Y/PY/NI to 2.5: Was there potential for a substantial impact (on the estimated effect of intervention) of analysing participants in the wrong group? | NA / Y / PY / PN / N / NI |  |
|  | **Risk of bias judgement** | Low / High / Some concerns |  |
|  | Optional: What is the predicted direction of bias due to deviations from intended interventions? | Favours experimental / Favours comparator / Towards null /Away from null / Unpredictable |  |
| **Bias due to missing outcome data** | 3.1 Were outcome data available for all, or nearly all, participants randomized? | Y / PY / PN / N / NI |  |
|  | 3.2 If N/PN/NI to 3.1: Are the proportions of missing outcome data and reasons for missing outcome data similar across intervention groups? | NA / Y / PY / PN / N / NI |  |
|  | 3.3 If N/PN/NI to 3.1: Is there evidence that results were robust to the presence of missing outcome data? | NA / Y / PY / PN / N / NI |  |
|  | **Risk of bias judgement** | Low / High / Some concerns |  |
|  | Optional: What is the predicted direction of bias due to missing outcome data? | Favours experimental / Favours comparator / Towards null /Away from null / Unpredictable |  |
| **Bias in measurement of the outcome** | 4.1 Were outcome assessors aware of the intervention received by study participants? | Y / PY / PN / N / NI |  |
|  | 4.2 If Y/PY/NI to 4.1: Was the assessment of the outcome likely to be influenced by knowledge of intervention received? | NA / Y / PY / PN / N / NI |  |
|  | **Risk of bias judgement** | Low / High / Some concerns |  |
|  | Optional: What is the predicted direction of bias due to measurement of the outcome? | Favours experimental / Favours comparator / Towards null /Away from null / Unpredictable |  |
| **Bias in selection of the reported result** | Are the reported outcome data likely to have been selected, on the basis of the results, from... |  |  |
|  | 5.1. ... multiple outcome measurements (e.g. scales, definitions, time points) within the outcome domain? | Y / PY / PN / N / NI |  |
|  | 5.2 ... multiple analyses of the data? | Y / PY / PN / N / NI |  |
|  | **Risk of bias judgement** | Low / High / Some concerns |  |
|  | Optional: What is the predicted direction of bias due to selection of the reported result? | Favours experimental / Favours comparator / Towards null /Away from null / Unpredictable |  |
| **Overall bias** | **Risk of bias judgement** | Low / High / Some concerns |  |
|  | Optional:  What is the overall predicted direction of bias for this outcome? | Favours experimental / Favours comparator / Towards null /Away from null / Unpredictable |  |

#### Risk of bias assessment for a cluster randomised parallel group trial^[[2]](#footnote-2)^

| **Bias domain** | **Signalling questions** | **Response options** | **Description/Support for judgement** |
| --- | --- | --- | --- |
| **Bias arising from the randomization process** | 1a.1 Was the allocation sequence random? | Y / PY / PN / N / NI |  |
|  | 1a.2 Is it likely that the allocation sequence was subverted? | Y / PY / PN / N / NI |  |
|  | 1a.3 Were there baseline imbalances that suggest a problem with the randomization process? | Y / PY / PN / N / NI |  |
|  | **Risk of bias judgement** | Low / High / Some concerns |  |
|  | Optional: What is the predicted direction of bias arising from the randomization process? | Favours experimental / Favours comparator / Towards null /Away from null / Unpredictable |  |
| **Bias arising from the timing of identification and recruitment of individual participants in relation to timing of randomization** | 1b.1 Were all the individual participants identified before randomization of clusters (and if the trial specifically recruited patients were they all recruited before randomization of clusters)? | Y / PY / PN / N / NI |  |
|  | 1b.2 If N/PN/NI to 1b.1: Is it likely that selection of individual participants was affected by knowledge of the intervention? | NA / Y / PY / PN / N / NI |  |
|  | 1b.3 Were there baseline imbalances that suggest differential identification or recruitment of individual participants between arms? | Y / PY / PN / N / NI |  |
|  | **Risk of bias judgement** | Low / High / Some concerns |  |
|  | Optional: What is the predicted direction of bias arising from the timing of identification and recruitment of individual participants? | Favours experimental / Favours comparator / Towards null /Away from null / Unpredictable |  |
| **Bias due to deviations from intended interventions** | 2.1a Were participants aware that they were in a trial? | Y / PY / PN / N / NI |  |
|  | 2.1b If Y/PY/NI to 2.1a: Were participants aware of their assigned intervention during the trial? | NA / Y / PY / PN / N / NI |  |
|  | 2.2. Were carers and trial personnel aware of participants' assigned intervention during the trial? | Y / PY / PN / N / NI |  |
|  | 2.3. If Y/PY/NI to 2.1 or 2.2: Were there deviations from the intended intervention beyond what would be expected in usual practice? | NA / Y / PY / PN / N / NI |  |
|  | 2.4. If Y/PY to 2.3: Were these deviations from intended intervention unbalanced between groups *and* likely to have affected the outcome? | NA / Y / PY / PN / N / NI |  |
|  | 2.5a Were any clusters analysed in a group different from the one to which they were assigned? | Y / PY / PN / N / NI |  |
|  | 2.5b Were any participants analysed in a group different from the one to which their original cluster was randomized? | Y / PY / PN / N / NI |  |
|  | 2.6 If Y/PY/NI to 2.5: Was there potential for a substantial impact (on the estimated effect of intervention) of analysing participants in the wrong group? | NA / Y / PY / PN / N / NI |  |
|  | **Risk of bias judgement** | Low / High / Some concerns |  |
|  | Optional: What is the predicted direction of bias due to deviations from intended interventions? | Favours experimental / Favours comparator / Towards null /Away from null / Unpredictable |  |
| **Bias due to missing outcome data** | 3.1a Were outcome data available for all, or nearly all, clusters randomized? | Y / PY / PN / N / NI |  |
|  | 3.1b Were outcome data available for all, or nearly all, participants within clusters? |  |  |
|  | 3.2 If N/PN/NI to 3.1a or 3.1b: Are the proportions of missing outcome data and reasons for missing outcome data similar across intervention groups? | NA / Y / PY / PN / N / NI |  |
|  | 3.3 If N/PN/NI to 3.1a or 3.1b: Is there evidence that results were robust to the presence of missing outcome data? | NA / Y / PY / PN / N / NI |  |
|  | **Risk of bias judgement** | Low / High / Some concerns |  |
|  | Optional: What is the predicted direction of bias due to missing outcome data? | Favours experimental / Favours comparator / Towards null /Away from null / Unpredictable |  |
| **Bias in measurement of the outcome** | 4.1a Were outcome assessors aware that a trial was taking place? | Y / PY / PN / N / NI |  |
|  | 4.1b If Y/PY/NI to 4.1: Were outcome assessors aware of the intervention received by study participants? | NA / Y / PY / PN / N / NI |  |
|  | 4.2 If Y/PY/NI to 4.1: Was the assessment of the outcome likely to be influenced by knowledge of intervention received? | NA / Y / PY / PN / N / NI |  |
|  | **Risk of bias judgement** | Low / High / Some concerns |  |
|  | Optional: What is the predicted direction of bias due to measurement of the outcome? | Favours experimental / Favours comparator / Towards null /Away from null / Unpredictable |  |
| **Bias in selection of the reported result** | Are the reported outcome data likely to have been selected, on the basis of the results, from... |  |  |
|  | 5.1. ... multiple outcome measurements (e.g. scales, definitions, time points) within the outcome domain? | Y / PY / PN / N / NI |  |
|  | 5.2 ... multiple analyses of the data? | Y / PY / PN / N / NI |  |
|  | **Risk of bias judgement** | Low / High / Some concerns |  |
|  | Optional: What is the predicted direction of bias due to selection of the reported result? | Favours experimental / Favours comparator / Towards null /Away from null / Unpredictable |  |
| **Overall bias** | **Risk of bias judgement** | Low / High / Some concerns |  |
|  | Optional:  What is the overall predicted direction of bias for this outcome? | Favours experimental / Favours comparator / Towards null /Away from null / Unpredictable |  |

### Risk of bias in cohort or case-control studies^[[3]](#footnote-3)^

#### Specify a target randomized trial specific to the study

| Design | Individually randomized / Cluster randomized / Matched (e.g. cross-over) |
| --- | --- |
| Participants |  |
| Experimental intervention |  |
| Comparator |  |

#### Is your aim for this study…?

| □ | to assess the effect of *assignment to* intervention |
| --- | --- |
| □ | to assess the effect of *starting and adhering to* intervention |

#### Specify the outcome

Specify which outcome is being assessed for risk of bias (typically from among those earmarked for the Summary of Findings table). Specify whether this is a proposed benefit or harm of intervention.

|  |
| --- |

#### Specify the numerical result being assessed

In case of multiple alternative analyses being presented, specify the numeric result (e.g. RR = 1.52 (95% CI 0.83 to 2.77) and/or a reference (e.g. to a table, figure or paragraph) that uniquely defines the result being assessed.

|  |
| --- |

#### Preliminary consideration of confounders

Complete a row for each important confounding domain (i) listed in the review protocol; and (ii) relevant to the setting of this particular study, or which the study authors identified as potentially important.

“Important” confounding domains are those for which, in the context of this study, adjustment is expected to lead to a clinically important change in the estimated effect of the intervention. “Validity” refers to whether the confounding variable or variables fully measure the domain, while “reliability” refers to the precision of the measurement (more measurement error means less reliability).

| **(i) Confounding domains listed in the review protocol** | | | | |
| --- | --- | --- | --- | --- |
| Confounding domain | Measured variable(s) | Is there evidence that controlling for this variable was unnecessary?* | Is the confounding domain measured validly and reliably by this variable (or these variables)? | OPTIONAL: Is failure to adjust for this variable (alone) expected to favour the experimental intervention or the comparator? |
|  |  |  | Yes / No / No information | Favour experimental / Favour comparator / No information |
|  |  |  |  |  |
|  |  |  |  |  |
|  |  |  |  |  |
|  |  |  |  |  |
|  |  |  |  |  |
|  |  |  |  |  |
|  |  |  |  |  |
|  |  |  |  |  |

| **(ii) Additional confounding domains relevant to the setting of this particular study, or which the study authors identified as important** | | | | |
| --- | --- | --- | --- | --- |
| Confounding domain | Measured variable(s) | Is there evidence that controlling for this variable was unnecessary?* | Is the confounding domain measured validly and reliably by this variable (or these variables)? | OPTIONAL: Is failure to adjust for this variable (alone) expected to favour the experimental intervention or the comparator? |
|  |  |  | Yes / No / No information | Favour experimental / Favour comparator / No information |
|  |  |  |  |  |
|  |  |  |  |  |
|  |  |  |  |  |
|  |  |  |  |  |
|  |  |  |  |  |
|  |  |  |  |  |
|  |  |  |  |  |
|  |  |  |  |  |
|  |  |  |  |  |

* In the context of a particular study, variables can be demonstrated not to be confounders and so not included in the analysis: (a) if they are not predictive of the outcome; (b) if they are not predictive of intervention; or (c) because adjustment makes no or minimal difference to the estimated effect of the primary parameter. Note that “no statistically significant association” is not the same as “not predictive”.

#### Preliminary consideration of co-interventions

Complete a row for each important co-intervention (i) listed in the review protocol; and (ii) relevant to the setting of this particular study, or which the study authors identified as important.

“Important” co-interventions are those for which, in the context of this study, adjustment is expected to lead to a clinically important change in the estimated effect of the intervention.

| **(i) Co-interventions listed in the review protocol** | | |
| --- | --- | --- |
| Co-intervention | Is there evidence that controlling for this co-intervention was unnecessary (e.g. because it was not administered)? | Is presence of this co-intervention likely to favour outcomes in the experimental intervention or the comparator |
|  |  | Favour experimental / Favour comparator / No information |
|  |  | Favour experimental / Favour comparator / No information |
|  |  | Favour experimental / Favour comparator / No information |
|  |  | Favour experimental / Favour comparator / No information |

| **(ii) Additional co-interventions relevant to the setting of this particular study, or which the study authors identified as important** | | |
| --- | --- | --- |
| Co-intervention | Is there evidence that controlling for this co-intervention was unnecessary (e.g. because it was not administered)? | Is presence of this co-intervention likely to favour outcomes in the experimental intervention or the comparator |
|  |  | Favour experimental / Favour comparator / No information |
|  |  | Favour experimental / Favour comparator / No information |
|  |  | Favour experimental / Favour comparator / No information |
|  |  | Favour experimental / Favour comparator / No information |

#### Risk of bias assessment

Responses underlined in green are potential markers for low risk of bias, and responses in red are potential markers for a risk of bias. Where questions relate only to sign posts to other questions, no formatting is used.

|  | **Signalling questions** | **Description** | **Response options** |
| --- | --- | --- | --- |
| **Bias due to confounding** | | | |
|  | 1.1 Is there potential for confounding of the effect of intervention in this study?  **If N/PN to 1.1:** the study can be considered to be at low risk of bias due to confounding and no further signalling questions need be considered |  | Y / PY / PN / N |
|  | **If Y/PY to 1.1**: determine whether there is a need to assess time-varying confounding: |  |  |
|  | 1.2. Was the analysis based on splitting participants’ follow up time according to intervention received?  **If N/PN**, answer questions relating to baseline confounding (1.4 to 1.6)  **If Y/PY**, go to question 1.3. |  | NA / Y / PY / PN / N / NI |
|  | 1.3. Were intervention discontinuations or switches likely to be related to factors that are prognostic for the outcome?  **If N/PN**, answer questions relating to baseline confounding (1.4 to 1.6)  **If Y/PY**, answer questions relating to both baseline and time-varying confounding (1.7 and 1.8) |  | NA / Y / PY / PN / N / NI |

|  | **Questions relating to baseline confounding only** | | |
| --- | --- | --- | --- |
|  | 1.4. Did the authors use an appropriate analysis method that controlled for all the important confounding domains? |  | NA / Y / PY / PN / N / NI |
|  | 1.5. **If Y/PY to 1.4**: Were confounding domains that were controlled for measured validly and reliably by the variables available in this study? |  | NA / Y / PY / PN / N / NI |
|  | 1.6. Did the authors control for any post-intervention variables that could have been affected by the intervention? |  | NA / Y / PY / PN / N / NI |
|  | **Questions relating to baseline and time-varying confounding** | |  |
|  | 1.7. Did the authors use an appropriate analysis method that controlled for all the important confounding domains and for time-varying confounding? |  | NA / Y / PY / PN / N / NI |
|  | 1.8. **If Y/PY to 1.7**: Were confounding domains that were controlled for measured validly and reliably by the variables available in this study? |  | NA / Y / PY / PN / N / NI |
|  | **Risk of bias judgement** |  | Low / Moderate / Serious / Critical / NI |
|  | Optional: What is the predicted direction of bias due to confounding? |  | Favours experimental / Favours comparator / Unpredictable |

| **Bias in selection of participants into the study** | | | |
| --- | --- | --- | --- |
|  | 2.1. Was selection of participants into the study (or into the analysis) based on participant characteristics observed after the start of intervention?  **If N/PN to 2.1:** go to 2.4 |  | Y / PY / PN / N / NI |
|  | 2.2. **If Y/PY to 2.1**: Were the post-intervention variables that influenced selection likely to be associated with intervention?  2.3 **If Y/PY to 2.2**: Were the post-intervention variables that influenced selection likely to be influenced by the outcome or a cause of the outcome? |  | NA / Y / PY / PN / N / NI  NA / Y / PY / PN / N / NI |
|  | 2.4. Do start of follow-up and start of intervention coincide for most participants? |  | Y / PY / PN / N / NI |
|  | 2.5. **If Y/PY to 2.2 and 2.3, or N/PN to 2.4**: Were adjustment techniques used that are likely to correct for the presence of selection biases? |  | NA / Y / PY / PN / N / NI |
|  | **Risk of bias judgement** |  | Low / Moderate / Serious / Critical / NI |
|  | Optional: What is the predicted direction of bias due to selection of participants into the study? |  | Favours experimental / Favours comparator / Towards null /Away from null / Unpredictable |

| **Bias in classification of interventions** | | | |
| --- | --- | --- | --- |
|  | 3.1 Were intervention groups clearly defined? |  | Y / PY / PN / N / NI |
|  | 3.2 Was the information used to define intervention groups recorded at the start of the intervention? |  | Y / PY / PN / N / NI |
|  | 3.3 Could classification of intervention status have been affected by knowledge of the outcome or risk of the outcome? |  | Y / PY / PN / N / NI |
|  | **Risk of bias judgement** |  | Low / Moderate / Serious / Critical / NI |
|  | Optional: What is the predicted direction of bias due to classification of interventions? |  | Favours experimental / Favours comparator / Towards null /Away from null / Unpredictable |

| **Bias due to deviations from intended interventions** | | | |
| --- | --- | --- | --- |
|  | **If your aim for this study is to assess the effect of assignment to intervention, answer questions 4.1 and 4.2** | |  |
|  | 4.1. Were there deviations from the intended intervention beyond what would be expected in usual practice? |  | Y / PY / PN / N / NI |
|  | 4.2. **If Y/PY to 4.1**: Were these deviations from intended intervention unbalanced between groups *and* likely to have affected the outcome? |  | NA / Y / PY / PN / N / NI |
|  | **If your aim for this study is to assess the effect of starting and adhering to intervention, answer questions 4.3 to 4.6** | |  |
|  | 4.3. Were important co-interventions balanced across intervention groups? |  | Y / PY / PN / N / NI |
|  | 4.4. Was the intervention implemented successfully for most participants? |  | Y / PY / PN / N / NI |
|  | 4.5. Did study participants adhere to the assigned intervention regimen? |  | Y / PY / PN / N / NI |
|  | 4.6. **If N/PN to 4.3, 4.4 or 4.5**: Was an appropriate analysis used to estimate the effect of starting and adhering to the intervention? |  | NA / Y / PY / PN / N / NI |
|  | **Risk of bias judgement** |  | Low / Moderate / Serious / Critical / NI |
|  | Optional: What is the predicted direction of bias due to deviations from the intended interventions? |  | Favours experimental / Favours comparator / Towards null /Away from null / Unpredictable |

| **Bias due to missing data** | | | |
| --- | --- | --- | --- |
|  | 5.1 Were outcome data available for all, or nearly all, participants? |  | Y / PY / PN / N / NI |
|  | 5.2 Were participants excluded due to missing data on intervention status? |  | Y / PY / PN / N / NI |
|  | 5.3 Were participants excluded due to missing data on other variables needed for the analysis? |  | Y / PY / PN / N / NI |
|  | 5.4 **If PN/N to 5.1, or Y/PY to 5.2 or 5.3**: Are the proportion of participants and reasons for missing data similar across interventions? |  | NA / Y / PY / PN / N / NI |
|  | 5.5 **If PN/N to 5.1, or Y/PY to 5.2 or 5.3**: Is there evidence that results were robust to the presence of missing data? |  | NA / Y / PY / PN / N / NI |
|  | **Risk of bias judgement** |  | Low / Moderate / Serious / Critical / NI |
|  | Optional: What is the predicted direction of bias due to missing data? |  | Favours experimental / Favours comparator / Towards null /Away from null / Unpredictable |

| **Bias in measurement of outcomes** | | | |
| --- | --- | --- | --- |
|  | 6.1 Could the outcome measure have been influenced by knowledge of the intervention received? |  | Y / PY / PN / N / NI |
|  | 6.2 Were outcome assessors aware of the intervention received by study participants? |  | Y / PY / PN / N / NI |
|  | 6.3 Were the methods of outcome assessment comparable across intervention groups? |  | Y / PY / PN / N / NI |
|  | 6.4 Were any systematic errors in measurement of the outcome related to intervention received? |  | Y / PY / PN / N / NI |
|  | **Risk of bias judgement** |  | Low / Moderate / Serious / Critical / NI |
|  | Optional: What is the predicted direction of bias due to measurement of outcomes? |  | Favours experimental / Favours comparator / Towards null /Away from null / Unpredictable |

| **Bias in selection of the reported result** | | | |
| --- | --- | --- | --- |
|  | Is the reported effect estimate likely to be selected, on the basis of the results, from... |  |  |
|  | 7.1. ... multiple outcome *measurements* within the outcome domain? |  | Y / PY / PN / N / NI |
|  | 7.2 ... multiple *analyses* of the intervention-outcome relationship? |  | Y / PY / PN / N / NI |
|  | 7.3 ... different *subgroups*? |  | Y / PY / PN / N / NI |
|  | **Risk of bias judgement** |  | Low / Moderate / Serious / Critical / NI |
|  | Optional: What is the predicted direction of bias due to selection of the reported result? |  | Favours experimental / Favours comparator / Towards null /Away from null / Unpredictable |

| **Overall bias** | | | |
| --- | --- | --- | --- |
|  | **Risk of bias judgement** |  | Low / Moderate / Serious / Critical / NI |
|  | Optional: What is the overall predicted direction of bias for this outcome? |  | Favours experimental / Favours comparator / Towards null /Away from null / Unpredictable |

### Risk of bias in cross-sectional studies^[[4]](#footnote-4)^

|  |  | **Yes** | **No** | **Do not know/ comment** |
| --- | --- | --- | --- | --- |
|  | ***Introduction*** |  |  |  |
| 1 | Were the aims/objectives of the study clear? |  |  |  |
|  | ***Methods*** |  |  |  |
| 2 | Was the study design appropriate for the stated aim(s)? |  |  |  |
| 3 | Was the sample size justified? |  |  |  |
| 4 | Was the target/reference population clearly defined? (Is it clear who the research was about?) |  |  |  |
| 5 | Was the sample frame taken from an appropriate population base so that it closely represented the target/reference population under investigator? |  |  |  |
| 6 | Was the selection process likely to select subjects/participants that were representative of the target/reference population under investigation? |  |  |  |
| 7 | Were measures undertaken to address and categorise non-responders? |  |  |  |
| 8 | Were the risk factor and outcome variables measured appropriate to the aims of the study? |  |  |  |
| 9 | Were the risk factor and outcome variables measured correctly using instruments/measurements that had been trialled, piloted or published previously? |  |  |  |
| 10 | Is it clear what was used to determine statistical significance and/or precision estimates? (eg. p-values, confidence intervals) |  |  |  |
| 11 | Were the methods (including statistical methods) sufficiently described to enable them to be repeated? |  |  |  |
|  | ***Results*** |  |  |  |
| 12 | Were the basic data adequately described? |  |  |  |
| 13 | Does the response rate raise concerns about non-response bias? |  |  |  |
| 14 | If appropriate, was information about non-responders described? |  |  |  |
| 15 | Were the results internally consistent? |  |  |  |
| 16 | Were the results presented for all the analyses described in the methods? |  |  |  |
|  | ***Discussion*** |  |  |  |
| 17 | Were the authors’ discussions and conclusions justified by the results? |  |  |  |
| 18 | Were the limitations of the study discussed? |  |  |  |
|  | ***Other*** |  |  |  |
| 19 | Were there any funding sources or conflicts of interest that may affect the authors’ interpretation of the results? |  |  |  |
| 20 | Was ethical approval or consent of participants attained? |  |  |  |

### Section 9: Notes

## Form 2: Qualitative data extraction form

### Section 1: General information

| Date form completed: |  |
| --- | --- |
| Study ID: |  |
| Citation: |  |
| Year: |  |
| Article type: |  |
| Author contact details for study: |  |
| Further information required: |  |
| Correspondence with authors: |  |
| Any additional unpublished data supplied? |  |
| Other information & notes |  |

### Section 2: Study it refers to

|  | **Instructions** | **Data** |
| --- | --- | --- |
| Type of study | *Eg. RCT, cohort, systematic review. If RCT, describe phase and design.* |  |
| Study disease area |  |  |
| Geographical location |  |  |
| Study population | *Describe age, gender, ethnicity and other key characteristics that may be relevant* |  |
| Study interventions | *If applicable* |  |
| Number of participants | *By group, if applicable* |  |
| When study took place |  |  |
| Funding source |  |  |
| Headline finding |  |  |

### Section 3: Study methods

| Aim of study |  |
| --- | --- |
| Theoretical background of study |  |
| Sampling approach |  |
| Data collection methods |  |
| Data analysis approach |  |

### Section 4: Participants in this study of communications

|  | **Instructions** | **Data** |
| --- | --- | --- |
| Who data was collected from | *Description. Professional or lay?* |  |
| Geographic location | *Where this study of communications took place* |  |
| Setting | *Where this study of communications took place.* |  |
| Method of recruitment of participants |  |  |
| Age: range |  |  |
| Gender |  |  |
| Ethnicity |  |  |
| Number of participants approached |  |  |
| Number who refused to take part |  |  |
| Number excluded |  |  |

### Section 5: Approach to communications studied

|  | **Instructions** | **Approach** | **Control (if applicable)** |
| --- | --- | --- | --- |
| Approach name |  |  |  |
| Approach aim & rationale | *Describe any theory and evidence base supporting the approach* |  |  |
| Tool(s)/Materials | *Describe the content, format(s) or media, source of materials (if possible, where they can be accessed), and any other information relevant to the physical or information materials provided to participants or in training providers of the approach.* |  |  |
| Procedures |  |  |  |
| Co-interventions |  |  |  |
| Mode of delivery | *eg. face-to-face / distant; individual/group* |  |  |
| Who delivered the approach? | *Include description of any specific training given to providers to deliver the approach, numbers of providers, professional background, specific pre-existing skills or experience required, quality of any specific training received to deliver the intervention, and any measures of competence or consistency in delivering the approach recorded before or during the study.* |  |  |
| Where was communication provided? | *(eg country, type of clinic, primary or hospital care).* |  |  |
| When and how often or much of the approach was provided? | *Describe how the approach was delivered, such as stages, timing, frequency, number of sessions, intensity and duration of approach delivery.* |  |  |
| Was the approach tailored? | *If the approach was meant to be tailored or personalised in the course of the study, describe the rationale for this and the major features of what was done - such as:*   - *how?* - *why?* - *when? and* - *what?*   *was done to tailor the approach.*  *If particular decision rules were used to determine when or how to tailor the approach details should be provided.* |  |  |
| Was the approach modified or adapted? | *If the approach was changed during the study, this should be described* |  |  |
| How well was the approach delivered? | *Assessment of fidelity: if approach fidelity was assessed, describe the extent to which the intervention was delivered as intended.* |  |  |

### Section 6: Findings and interpretation

| Key themes identified in the study |  |
| --- | --- |
| Data extracts related to the key themes |  |
| Interpretation |  |
| Recommendations made by author |  |

### Section 7: Risk of bias^[[5]](#footnote-5)^

| **Question** | **Hints** | **Assessment** | **Comments** |
| --- | --- | --- | --- |
| **Section A: Are the results of valid?** | | | |
| **1. Was there a clear statement of the aims of the research?** | - *What was the goal of the research?* - *Why it was thought important* - *Its relevance* | Yes  Can’t Tell  No |  |
| **2. Is a qualitative methodolgy appropriate?** | - *Does the research seek to interpret or illuminate the actions and/or subjective experiences of research participants?* - *Is qualitative research the right methodology for addressing the research goal?* | Yes  Can’t Tell  No |  |
| **Is it worth continuing?** | | | |
| **3. Was the research design appropriate to address the aims of the research?** | - *Has the researcher justified the research design?* | Yes  Can’t Tell  No |  |
| **4. Was the recruitment strategy appropriate to the aims of the research?** | - *Does the researcher explain how the participants were selected?* - *Do they explain why the participants selected were the most appropriate to provide access to the type of knowledge sought by the study?* - *Is there any discussion around recruitment?* | Yes  Can’t Tell  No |  |
| **5. Was the data collected in a way that addressed the research issue?** | - *Was the setting for data collection justified?* - *Is it clear how data were collected?* - *Has the researcher justified the methods chosen?* - *Has the researcher made the methods explicit* - *If methods were modified during the study, has the researcher explained why and how?* - *Is the form of data clear?* - *Has researcher discussed saturation of data?* | Yes  Can’t Tell  No |  |
| **6. Has the relationship between researcher and participants been adequately considered?** | - *Has the researcher critically examined their own role, potential bias and influence during formulation of research question, and data collection* - *How did the researcher respond to events during the study, and did they consider the implications of any changes in the research design?* | Yes  Can’t Tell  No |  |
| **What are the results?** | | | |
| **7. Have ethical issues been taken into consideration?** | - *Do they give details of how the research was explained to participants?* - *Do they discuss issues raised by the study?* - *Was approval sought from the ethics committee?* | Yes  Can’t Tell  No |  |
| **8. Was the data analysis sufficiently rigorous?** | - *Is there an indepth description of the analysis process?* - *Is it clear how categories/themes were derived from the data?* - *Does the research explain how the data presented were selected from the original sample?* - *Is sufficient data presented to support the findings?* - *To what extent are contradictory data taken into account?* - *Does the researcher critically examine their own role, bias and influence during analysis?* | Yes  Can’t Tell  No |  |
| **9. Is there a clear statement of findings?** | - *Are the findings explicit?* - *Is there adequate discussion of the evidence for and against the researcher’s arguments* - *Does the researcher discuss the credibility of their findings?* - *Are the findings discussed in relation to the original research question?* | Yes  Can’t Tell  No |  |

### Section 9: Notes

1. RoB 2.0 template for individual RCTs [↑](#footnote-ref-1)
2. RoB 2.0 for cluster randmised trial when interest is in the effect of assignment to intervention [↑](#footnote-ref-2)
3. The Risk of Bias in Non-randomized Studies – of Interventions (ROBINS-1) assessment tool <https://sites.google.com/site/riskofbiastool/welcome/home/current-version-of-robins-i/robins-i-template-2016?authuser=0> [↑](#footnote-ref-3)
4. AXIS tool <https://bmjopen.bmj.com/content/6/12/e011458> [↑](#footnote-ref-4)
5. CASP Qualitative Checklist <https://casp-uk.net/wp-content/uploads/2018/03/CASP-Qualitative-Checklist-Download.pdf> [↑](#footnote-ref-5)
